# Artery reopening is required for the neurorestorative effects of angiotensin modulation after experimental stroke

**DOI:** 10.1186/s13231-016-0018-x

**Published:** 2016-04-27

**Authors:** Ahmed Alhusban, Anna Kozak, Wael Eldashan, Adviye Ergul, Susan C. Fagan

**Affiliations:** Charlie Norwood VA Medical Center, and Center for Pharmacy and Experimental Therapeutics, University of Georgia College of Pharmacy, and Georgia Regents University, Augusta, GA USA; Clinical Pharmacy Department, College of Pharmacy, Jordan University of Science and Technology, P.O. Box 3030, Irbid, 22110 Jordan

**Keywords:** Ischemic stroke, AT1 blockers, BDNF, Neuroprotection, Reperfusion

## Abstract

**Background:**

Blood flow restoration with fibrinolysis and thrombectomy is recommended to limit injury in stroke patients with proximal artery occlusion. Angiotensin receptor blockers have been shown to be neuroprotective in models of permanent and temporary occlusion, but the benefits on expression of trophic factors have been seen only when the artery is reopened. It is possible that early artery opening with endovascular intervention may increase the likelihood of identifying an effective combination therapy for patients.

**Methods:**

Normotensive male Wistar rats were subjected to mechanical middle cerebral artery occlusion (either temporary or permanent), followed by randomization to receive candesartan (0.3 mg/kg IV) or saline. Functional outcome, infarct size, and biochemical changes were assessed 24 h after ischemia induction.

**Results:**

Lack of reperfusion blunted candesartan induced neuroprotection (p < 0.05) and reduced the improvement of functional outcome (p < 0.05). With reperfusion, candesartan increased mature BDNF expression in the contralateral hemisphere (p < 0.05) and activated prosurvival (Akt-GSK3-β) signaling (p < 0.05). Without reperfusion, candesartan significantly reduced VEGF expression and MMP activation and increased NOGO A expression, creating an environment hostile to recovery.

**Conclusion:**

Candesartan induced pro-recovery effects are dependent on the presence of reperfusion.

## Background

Stroke is an acute neurologic disease and a leading cause of morbidity and mortality [1]. In the majority of cases, stroke results from a mechanical occlusion of one of the intracranial arteries [1]. This results in reduced cerebral blood flow below a threshold necessary for neuronal function resulting in neurologic dysfunction [2, 3]. Re-establishing blood flow to the ischemic area is essential to salvage metabolically stunted tissue [4, 5]. In fact, the only FDA approved drug for the acute management of stroke, alteplase, is intended to restore blood flow to the affected region [5]. Despite its effectiveness, the use of alteplase is limited by a 4.5 h time window and a long list of contraindications [5].

Endovascular thrombectomy offers a new potential to restore cerebral perfusion [6–10]. Data from recent clinical trials demonstrated the safety and efficacy of thrombectomy with a stent retriever in the management of stroke [6–10]. The beneficial effect was observed even when the intervention was performed up to 12 h after the onset of the stroke [9]. In addition to restoring cerebral perfusion, opening an occluded artery is expected to theoretically improve drug delivery to the affected brain region. This might enhance the neuroprotective and pro-recovery effects of pharmaceutical agents. Although theoretically plausible, it is still unknown whether opening occluded arteries would impact the neuroprotective and pro-recovery effects of pharmaceutical agents.

Angiotensin receptor blockers (ARBs) have been shown to reduce the extent of neuronal damage and improve outcome after experimental cerebral ischemia [11–19]. ARB-induced neurovascular protective and pro-recovery effects were found to be mediated through a number of mechanisms including cerebral blood flow restoration, antinflammatory, and antioxidant effects [11, 14, 15, 20–23]. Data from our lab showed the ability of ARBs to induce a proangiogenic effect after stroke [12, 18] and we subsequently implicated an increased expression of growth factors after stroke in their pro-recovery effects [12, 18, 24, 25]. Although ARB-induced neuroprotection has been observed in studies with reperfusion component as well as in permanent occlusion studies [11, 12, 14, 15, 20, 22], it is still unknown whether the neuroprotective effects of ARBs are modulated by the presence of reperfusion in a direct head to head comparison. In this investigation, our aim was to assess whether reperfusion modulates candesartan induced pro-recovery effects.

## Methods

### Animals

All animal procedures were approved by the Institutional Animal Care and Use Committee (IACUC) of the Charlie Norwood Veterans Affairs Medical Center (09-04-008). Male Wistar rats (280–300 g) were subjected to middle cerebral artery occlusion (MCAO) as described earlier [16, 18]. Briefly, the ventral side of the neck was shaved and probed with iodine and 70 % ethanol. A midline incision was made to expose neck blood vessels. The common carotid artery (CCA) and the external carotid artery (ECA) were isolated. After being isolated, the ECA was ligated, cauterized and a small incision was introduced in the ECA stub. A silicone coated filament was then introduced into the internal carotid artery (ICA) through the ECA stub and was pushed all the way to block the origin of the middle cerebral artery (MCA). After 3 h of occlusion, animals either underwent reperfusion by withdrawal of the filament or remained permanently occluded. At the same time, these animals were randomized to receive either candesartan (0.3 mg/kg) or saline. Animals were followed up for 24 h after occlusion when they were sacrificed by decapitation. In one set of animals, brains were harvested, sliced and stained with 5 % Triphenyl Tetrazolium Chloride (TTC) to assess infarct size and edema volume. In another set of animals, brains were harvested and flash frozen in liquid nitrogen for biochemical analysis.

### Behavioral outcome

Behavioral outcome was evaluated 24 h after MCAO using the modified Bederson score as described previously [16].

### Protein quantification

Brains were homogenized using 1X RIPA buffer supplemented with protease inhibitor cocktail, PMSF, and sodium orthovanidate. Protein content was determined using bicinchonic acid (BCA) method (Thermo-Scientific) and 30 μg proteins from each samples were loaded and separated on 4–20 % ready-made criterion gel (Bio-Rad). Proteins were transferred to nitrocellulose membranes and membranes were blocked with 5 % low fat milk in TBST (1 % tween in Tris-Buffered Saline). The membranes were probed with antiBDNF (1:250; Santa Cruz biotechnologies; Santa Cruz, CA, USA), TrkB (1:500, abcam; Cambridge, MA, USA), pGSK3-β (1:1000, Cell Signaling; Danvers, MA, USA), total GSK3-β (1:1000, Cell Signaling; Danvers, MA, USA), pAkt-473(1:1000, Cell Signaling; Danvers, MA, USA), pan Akt (1:1000, Cell Signaling; Danvers, MA, USA). Expression was quantified by measuring the optic density of the band relative to its cognate actin band using image J software.

VEGF quantification was performed using enzyme-linked immunosorbent assay kit (RayBiotech, Norcross, GA, USA) according to the manufacturer protocol. Briefly, brain homogenates (100 μl) were incubated overnight on antibody coated plate. Wells were washed and then incubated with a biotinylated secondary antibody then TMB substrate was added and spectrophotometric analysis was done. Signal was measured at 450 nm (BioTek, Winooski, VT, USA).

### MMP zymography

MMP zymography was performed as described in Kozak et al. [18]. Briefly, brain homogenates were electrophoretically separated on an acrylamide gel containing gelatin. Gelatinolytic activity was quantified by densitometric analysis (Gel-Pro v 3.1; Media Cybernetics, Carlsbad, Calif).

### Statistical analysis

Statistical significance was determined using student t test and two way ANOVA as appropriate. Statistical analyses were performed using GraphPad prism software (5.1). p < 0.05 was considered significant.

## Results

### Candesartan induces neuroprotection and improves functional outcome

**Fig. 1:**
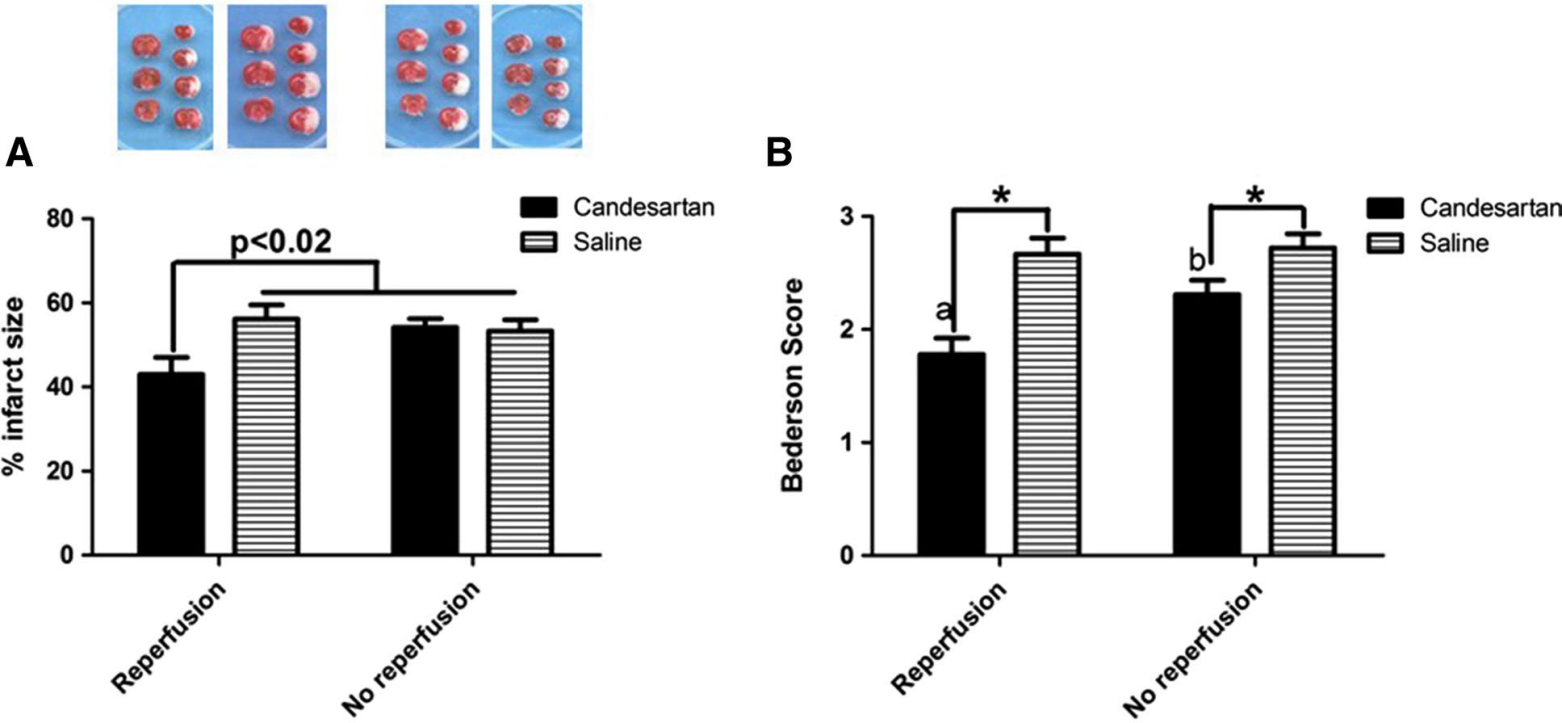
Reperfusion is essential for candesartan induced functional outcome improvement. Sub-hypotensive dose of candesartan conferred neuroprotective effect after stroke in a reperfusion dependent manner (**A**). In addition, the ability of candesartan to improve functional outcome is also reperfusion dependent (**B**). *p < 0.05, a, b pairs of means with* different letters* are significantly different from each other. n = 8–14 per group

Data from our lab and others have demonstrated the neuroprotective effect of candesartan at hypotensive doses in normotensive or hypertensive animals [11, 14, 18]. Accordingly, our interest shifted to assess whether sub-hypotensive doses of candesartan might induce a similar response in normotensive animals. To achieve this we used a dose of candesartan (0.3 mg/kg IV) that was demonstrated to have a minimal effect on blood pressure (BP) when administered at reperfusion [25]. In this study, candesartan 0.3 mg/kg reduced infarct size by 23 % (Fig. 1A) and improved the functional outcome as measured by modified Bederson score (Fig. 1B).

### Reperfusion is essential for ARB-induced neuroprotection but not for improving early functional outcome

**Fig. 2:**
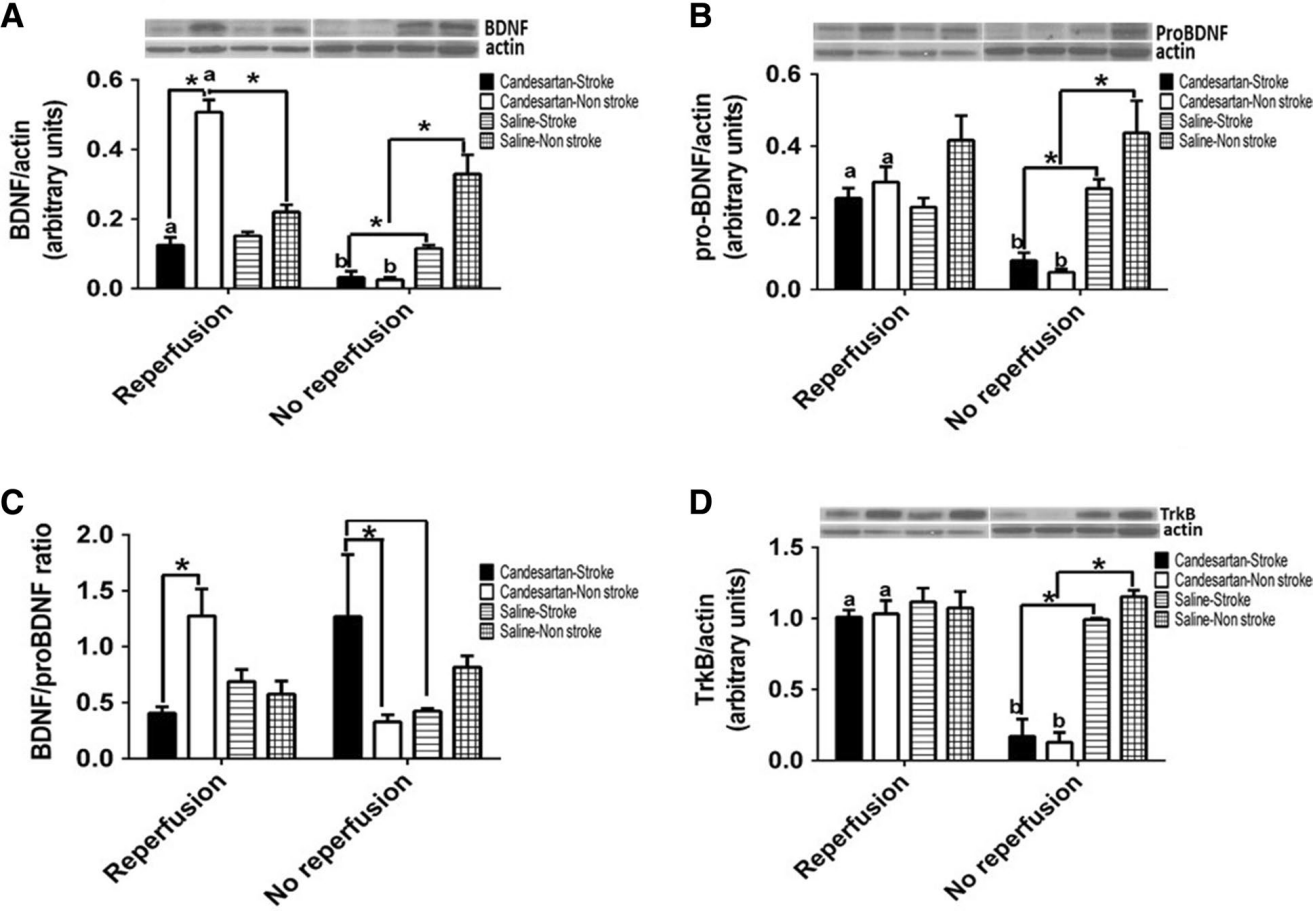
Reperfusion modulates the ability of candesartan to affect the expression of BDNF/TrkB system components. Candesartan increased mature BDNF expression in the contralateral hemisphere in a reperfusion dependent manner (**A**). The effect of reperfusion is most prominent in proBDNF (**B**), BDNF/proBDNF ratio (**C**), and TrkB (**D**) expression. Groups connected by* line* are significantly different from each other. a,b pairs of means with* different letters* are significantly different from each other. n = 4 per group

Despite being essential for salvaging neurons in the penumbra, reperfusion has also been shown to cause additional injury to the ischemic tissue [26, 27]. Our previous data demonstrated the effects of candesartan administered at the time of reperfusion on stroke outcome [12, 17]. In this investigation, we aimed at assessing whether reperfusion is necessary for candesartan induced neuroprotection and functional outcome improvement. Lack of reperfusion blunted candesartan induced neuroprotection (Fig. 2A), but candesartan induced improvement in functional outcome at 24 h was maintained (Fig. 1B).

### Candesartan modulates the expression of BDNF in a reperfusion dependent manner

BDNF has been shown to improve stroke outcome [28–31] and we showed that candesartan up-regulates BDNF expression in both Wistar and hypertensive (SHR) rats, with [25] and without stroke [24]. We then set out to determine whether reperfusion was necessary for this effect. After temporary MCAO, candesartan significantly increased mature BDNF in the contralateral hemisphere of candesartan treated animals as compared to the ipsilateral hemisphere (Fig. 2A). This increase in mature BDNF was accompanied by an increase in the mature to proBDNF ratio (Fig. 2C). In contrast to its effect in reperfused animals, candesartan reduced mature BDNF in both hemispheres of non-reperfused animals (Fig. 2A). Candesartan also reduced the expression of proBDNF levels in the brain (Fig. 2B). Candesartan did not alter the levels of proBDNF in reperfused animals (Fig. 2B).

### Candesartan modulates the expression of TrkB in a reperfusion dependent manner

BDNF induced neuroprotection is mediated through TrkB signaling [32, 33]. In reperfused brains, candesartan did not alter the expression of TrkB after stroke (Fig. 2D). Interestingly, candesartan administration significantly reduced TrkB expression in the absence of reperfusion (Fig. 2D).

### Candesartan up-regulates the pro-survival, Akt-GSK3-β axis

**Fig. 3:**
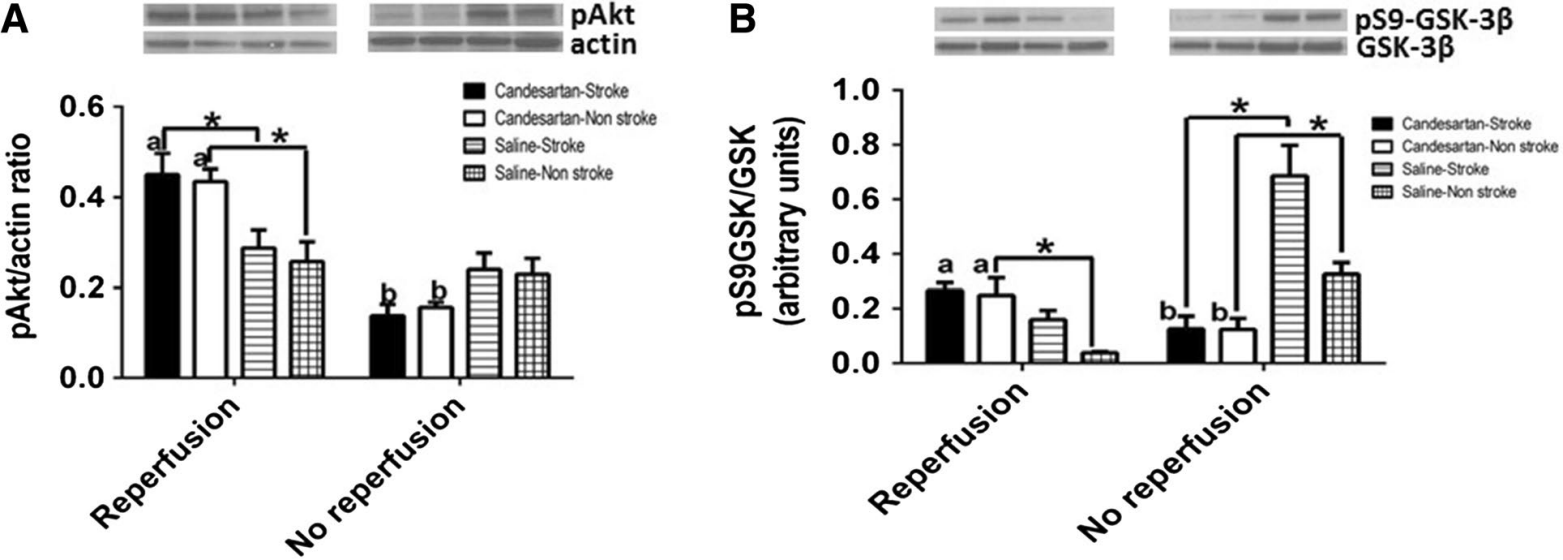
Reperfusion is necessary for candesartan induced activation of survival signaling and ER stress amelioration. Candesartan induced neuroprotection involves the activation of Akt (**A**) and GSK3-β signaling (**B**). Lack of reperfusion blunts the ability of candesartan to activate intracellular survival signals. Groups connected by line are significantly different from each other. a,b pairs of means with *different letters* are significantly different from each other. n = 4 per group

Recently, Guo et al. [13] demonstrated the involvement of Akt-GSK3-β activity in BDNF mediated neuroprotection conferred by endothelial cells. Previously, our data suggested the involvement of GSK3-β in candesartan induced up-regulation of BDNF expression in endothelial cells [24]. Candesartan administration significantly activated the Akt-GSK3-β axis in both ipsilateral and contralateral hemispheres (Fig. 3A, B, respectively).

### Candesartan induced activation of Akt-GSK3-β signaling is reperfusion dependent

Similar to our findings on candesartan induced neuroprotection, the activity of Akt-GSK3-β was reperfusion dependent. Lack of reperfusion blunted candesartan induced activation of Akt signaling (Fig. 3A) and GSK-3β inhibition (Fig. 3B).

### Candesartan reduced VEGF expression in non-reperfused brain after stroke

**Fig. 4:**
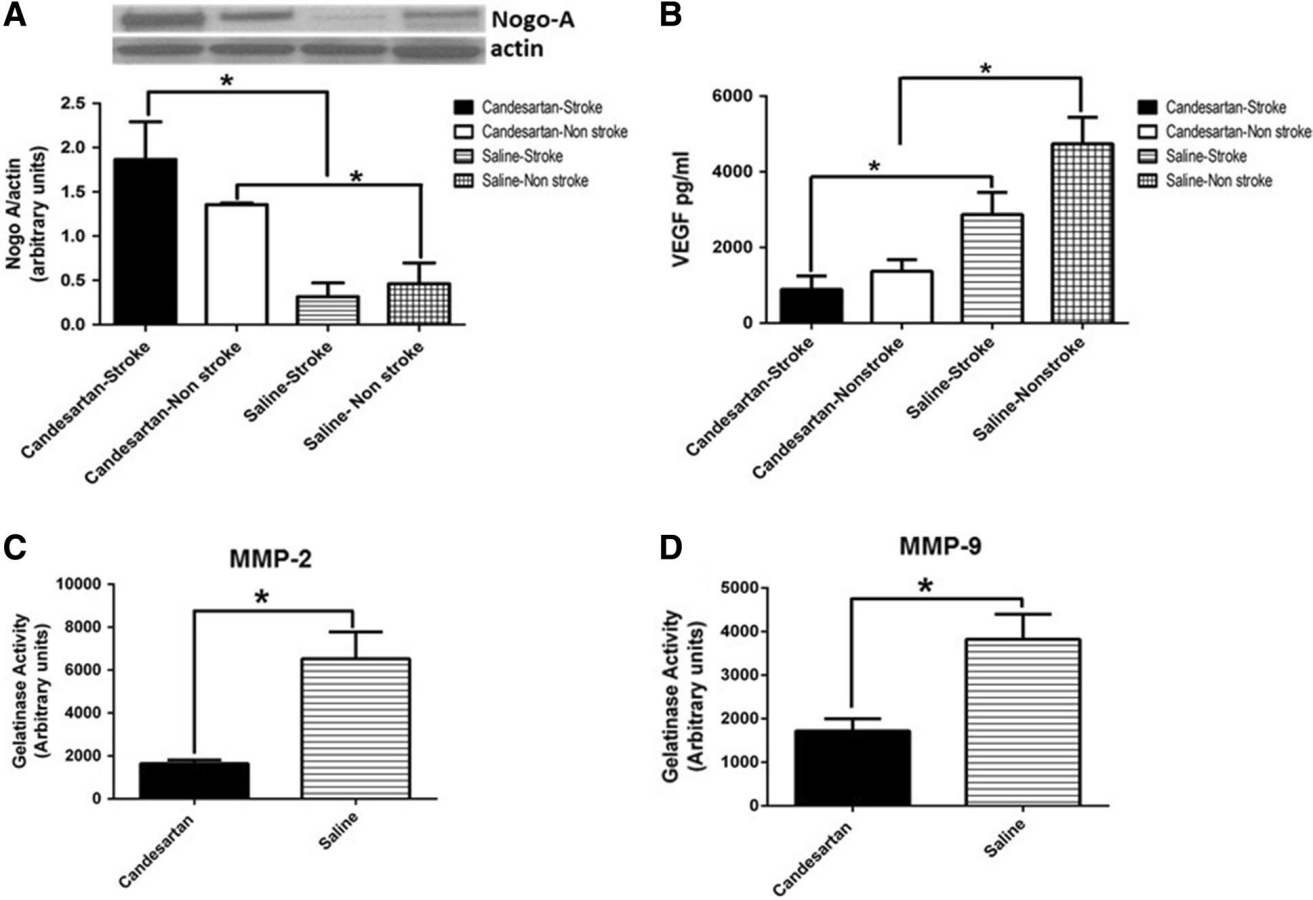
Candesartan increases the expression of Nogo-A, and reduced VEGF and MMPs activity in non-reperfused brains. Candesartan administration in animals exposed to permanent middle cerebral artery occlusion increased Nogo-A expression in both hemispheres (**A**). Candesartan administration significantly reduced VEGF expression (**B**) and MMP2 and 9 activities (**C** and **D**, respectively). Groups connected by* line* are significantly different from each other. **A**, **B** pairs of means with* different letters* are significantly different from each other. n = 4 per group

Data from our lab indicated the ability of candesartan to increase VEGF expression at both hypotensive [18] and sub-hypotensive doses [25]. These results were all derived from animals exposed to ischemia and reperfusion. It was unknown whether this effect of candesartan is reperfusion dependent. Accordingly, we quantified VEGF expression in non-reperfused animals. Candesartan treatment significantly reduced VEGF expression as compared to non-reperfused animals (Fig. 4B).

### Candesartan reduced MMP activity in non-reperfused animals after stroke

Kozak et al. [18] reported that candesartan induced prorecovery effect was associated with an increase in MMP activity in reperfused animals. It was unknown whether this effect is reperfusion dependent or not, however. Without reperfusion, candesartan administration had the opposite effect and significantly reduced both MMP2 (Fig. 4C) and MMP9 (Fig. 4D) activity after stroke.

### Candesartan increases Nogo-A expression in non-reperfused brain after stroke

To determine whether the observed effects of candesartan on prorecovery mediators in non-reperfused brain are due to an overall reduced protein expression, the expression of Nogo-A, a neurite outgrowth inhibitor, was quantified. Candesartan increased the expression of Nogo-A expression in non-reperfused brain (Fig. 4A).

## Discussion

Our results demonstrate, for the first time, the essential requirement of reperfusion for the full neurorestorative effects of candesartan. Our results demonstrate both neuroprotective and pro-recovery effects of candesartan, when administered at a dose of 0.3 mg/kg. Previously, we demonstrated a neuroprotective and pro-recovery effect of candesartan in normal and hypertensive rats [12, 16–19, 25] and at low and higher doses [25]. In a model of temporary cerebral ischemia, a single dose of candesartan administered at the time of reperfusion resulted in a prolonged pro-recovery effect [18]. This effect was associated with an increase in VEGF expression and MMP activity. In a follow-up study using the same dose, Guan et al. [17] demonstrated a reduction in infarct size in non-reperfused animals, but no effect of candesartan on VEGF expression, or MMP activity. These contradictory findings suggest a possible reperfusion dependence in some aspects of candesartan induced pro-recovery effect after stroke. We now demonstrate a stark contrast in the expression of pro-recovery mediators after candesartan in reperfused and non-reperfused animals. Accordingly, our data provides the first direct evidence that supports the notion raised by the others that candesartan induced neuroprotection and pro-recovery effect is reperfusion dependent.

Consistent with these findings, candesartan induced upregulation of mature BDNF levels was completely reversed in non reperfused brain. Mature BDNF content in the brain is determined by both de novo expression and ProBDNF processing into the mature form [33]. Our results suggest that in non-reperfused brain, AT1 blockade reduces de novo expression of BDNF as detected by a reduction in both pro and mature forms of BDNF. Similarly, candesartan significantly reduced the expression TrkB in non-reperfused brain. These findings are also consistently demonstrated in the activity of Akt-GSK-3β signaling axis which has been demonstrated to be involved in neuroprotection and improving functional outcome after stroke [13]. In reperfused brains, candesartan administration significantly increased Akt and GSK3-β phosphorylation. In contrast, candesartan administration in permanent stroke model significantly reduced Akt and GSK3-β activity.

A plausible explanation of these interesting findings is suggested by studies on penumbra development and cellular bioenergetics after ischemia. In an elegant work Mies et al. [34] reported a 55 ml/100 gm/min threshold for protein synthesis in the brain. Below this threshold, protein synthesis in the brain ceases. To account for this possibility, our interest shifted to quantify the expression of proteins known to be involved in worsening stroke outcome. One candidate protein in this setting is Nogo-A, which has been shown to worsen stroke outcome and to antagonize the effects of BDNF in neurons [35, 36]. If the observed reduction in BDNF and TrkB expression is due to stunted transcriptional machinery in non reperfused brain, Nogo-A expression would also be reduced. Interestingly, candesartan administration induced a robust increase in Nogo-A expression in non reperfused brain. Additionally, candesartan significantly reduced both VEGF expression and MMP2 and 9 in non reperfused brains. This finding suggests that candesartan induced reduction in BDNF and TrkB expression is not due to a mere synthetic machinery failure induced by a long duration of ischemia. In contrast, it suggests that lack of reperfusion reduces the expression or the bioavailability of essential mediators for candesartan induced neurorestoration. This finding may help explain the conflicting results of the ACCESS [37] and SCAST [38] clinical trials with regard to candesartan’s effect on stroke outcome, where reperfusion was not assured.

In conclusion, our results demonstrate a reperfusion dependent candesartan induced improved stroke outcome at sub-hypotensive doses. This neuroprotective and pro-recovery effect is mediated through an up-regulation of BDNF expression and the resulting activation of Akt-GSK3-β signaling.

## References

[1] Mozaffarian D (2015). Heart disease and stroke statistics–2015 update: a report from the American Heart Association. Circulation.

[2] Astrup J (1977). Cortical evoked potential and extracellular K+ and H+ at critical levels of brain ischemia. Stroke.

[3] Astrup J, Siesjo BK, Symon L (1981). Thresholds in cerebral ischemia—the ischemic penumbra. Stroke.

[4] Hacke W (1998). Randomised double-blind placebo-controlled trial of thrombolytic therapy with intravenous alteplase in acute ischaemic stroke (ECASS II). Second European-Australasian acute stroke study investigators. Lancet.

[5] Tissue plasminogen activator for acute ischemic stroke (1995). The National Institute of Neurological Disorders and Stroke rt-PA Stroke Study Group. N Engl J Med.

[6] Berkhemer OA (2015). A randomized trial of intraarterial treatment for acute ischemic stroke. N Engl J Med.

[7] Campbell BC (2015). Endovascular therapy for ischemic stroke with perfusion-imaging selection. N Engl J Med.

[8] Goyal M (2015). Randomized assessment of rapid endovascular treatment of ischemic stroke. N Engl J Med.

[9] Jovin TG (2015). Thrombectomy within 8 hours after symptom onset in ischemic stroke. N Engl J Med.

[10] Saver JL (2015). Stent-retriever thrombectomy after intravenous t-PA vs. t-PA alone in stroke. N Engl J Med.

[11] Dai WJ (1999). Blockade of central angiotensin AT(1) receptors improves neurological outcome and reduces expression of AP-1 transcription factors after focal brain ischemia in rats. Stroke.

[12] Guan W (2011). Vascular protection by angiotensin receptor antagonism involves differential VEGF expression in both hemispheres after experimental stroke. PLoS ONE.

[13] Guo S (2012). Vascular neuroprotection via TrkB- and Akt-dependent cell survival signaling. J Neurochem.

[14] Ito T (2002). Protection against ischemia and improvement of cerebral blood flow in genetically hypertensive rats by chronic pretreatment with an angiotensin II AT1 antagonist. Stroke.

[15] Zhou J (2006). AT1 receptor blockade regulates the local angiotensin II system in cerebral microvessels from spontaneously hypertensive rats. Stroke.

[16] Fagan SC (2006). Hypertension after experimental cerebral ischemia: candesartan provides neurovascular protection. J Hypertens.

[17] Guan W (2011). Acute treatment with candesartan reduces early injury after permanent middle cerebral artery occlusion. Transl Stroke Res.

[18] Kozak A (2009). Candesartan augments ischemia-induced proangiogenic state and results in sustained improvement after stroke. Stroke.

[19] Kozak W (2008). Vascular protection with candesartan after experimental acute stroke in hypertensive rats: a dose-response study. J Pharmacol Exp Ther.

[20] Engelhorn T (2004). The angiotensin II type 1-receptor blocker candesartan increases cerebral blood flow, reduces infarct size, and improves neurologic outcome after transient cerebral ischemia in rats. J Cereb Blood Flow Metab.

[21] Brdon J (2007). Comparison between early and delayed systemic treatment with candesartan of rats after ischaemic stroke. J Hypertens.

[22] Nishimura Y, Ito T, Saavedra JM (2000). Angiotensin II AT(1) blockade normalizes cerebrovascular autoregulation and reduces cerebral ischemia in spontaneously hypertensive rats. Stroke.

[23] Yamakawa H (2003). Normalization of endothelial and inducible nitric oxide synthase expression in brain microvessels of spontaneously hypertensive rats by angiotensin II AT1 receptor inhibition. J Cereb Blood Flow Metab.

[24] Alhusban A (2013). AT1 receptor antagonism is proangiogenic in the brain: BDNF a novel mediator. J Pharmacol Exp Ther.

[25] Ishrat T (2015). Low-dose candesartan enhances molecular mediators of neuroplasticity and subsequent functional recovery after ischemic stroke in rats. Mol Neurobiol.

[26] Pundik S, Xu K, Sundararajan S (2012). Reperfusion brain injury: focus on cellular bioenergetics. Neurology.

[27] Heiss WD (2012). The ischemic penumbra: how does tissue injury evolve?. Ann N Y Acad Sci.

[28] Muller HD (2008). Brain-derived neurotrophic factor but not forced arm use improves long-term outcome after photothrombotic stroke and transiently upregulates binding densities of excitatory glutamate receptors in the rat brain. Stroke.

[29] Schabitz WR (2004). Effect of brain-derived neurotrophic factor treatment and forced arm use on functional motor recovery after small cortical ischemia. Stroke.

[30] Schabitz WR (2007). Intravenous brain-derived neurotrophic factor enhances poststroke sensorimotor recovery and stimulates neurogenesis. Stroke.

[31] Mahmood A, Lu D, Chopp M (2004). Intravenous administration of marrow stromal cells (MSCs) increases the expression of growth factors in rat brain after traumatic brain injury. J Neurotrauma.

[32] Waterhouse EG, Xu B (2009). New insights into the role of brain-derived neurotrophic factor in synaptic plasticity. Mol Cell Neurosci.

[33] Marini AM (2004). Role of brain-derived neurotrophic factor and NF-kappaB in neuronal plasticity and survival: from genes to phenotype. Restor Neurol Neurosci.

[34] Mies G (1991). Ischemic thresholds of cerebral protein synthesis and energy state following middle cerebral artery occlusion in rat. J Cereb Blood Flow Metab.

[35] Kilic E (2010). Role of Nogo-A in neuronal survival in the reperfused ischemic brain. J Cereb Blood Flow Metab.

[36] Pernet V, Schwab ME (2012). The role of Nogo-A in axonal plasticity, regrowth and repair. Cell Tissue Res.

[37] Schrader J (2003). The ACCESS Study: evaluation of acute candesartan cilexetil therapy in stroke survivors. Stroke.

[38] Sandset EC (2011). The angiotensin-receptor blocker candesartan for treatment of acute stroke (SCAST): a randomised, placebo-controlled, double-blind trial. Lancet.

