# Erratum to: Artery reopening is required for the neurorestorative effects of angiotensin modulation after experimental stroke

**DOI:** 10.1186/s13231-016-0019-9

**Published:** 2016-06-14

**Authors:** Ahmed Alhusban, Anna Kozak, Wael Eldahshan, Adviye Ergul, Susan C. Fagan

**Affiliations:** Charlie Norwood VA Medical Center, and Center for Pharmacy and Experimental Therapeutics, University of Georgia College of Pharmacy, and Georgia Regents University, Augusta, GA USA; Clinical Pharmacy Department, College of Pharmacy, Jordan University of Science and Technology, P.O. Box 3030, Irbid, 22110 Jordan

## Erratum to: Exp & Trans Stroke Med (2016) 8:4 DOI 10.1186/s13231-016-0018-x

After publication of the original article [1], the corresponding author noted that one of the author’s names was spelt incorrectly. The correct name of the author (Wael Eldahshan) has been updated in the original article, and published in this Erratum for quick reference. We apologise for any confusion this may have caused.
